# Shrink Wrapped: Tales from Psychiatrists

**DOI:** 10.1192/pb.bp.116.055293

**Published:** 2017-08

**Authors:** Gary Cooney

There is something that feels a little otherworldly about *Shrink Wrapped*. As readers, we are invited to find our way into the psychiatrist's mind, to take our bearings from this anonymous collection of short anecdotes and reflections, written by psychiatrists, as a point of departure into understanding what it might mean to be a modern-day ‘shrink’. We find warm, affectionate and humorous musings that are abruptly punctured by sharper observations, memories painful to revisit, self-doubt, recrimination and a sense of ever-questioned legitimacy. It is an honest, undiluted look into the experiences of eight psychiatrists, the whispered, confessional moments that blurred the boundaries of their personal and professional lives.

There is a strength of variety to the collection and, at its best, it draws us into questioning ourselves: what kind of a psychiatrist have I become and how did I get here? Does the voice I'm reading remind me of my own thoughts or my own journey? There are, however, one or two weaker moments alongside: in its editorial efforts to be snapshot-succinct, it can sometimes feel frustratingly whimsical and underdeveloped, like a string of highly promising film trailers. There are also passages that veer a little close to what might be found in the reflective practice section of an online portfolio.

*Shrink Wrapped* is as intimate and conversational as a cup of tea with a close colleague. It considers our own professional self-consciousness, ‘the navel-gazing and self-doubt’ that besiege our specialty, and takes a frank, unabashed look at the inevitable cross-pollination of our work and life experiences. The collection provides the reader with moments of piercing honesty, such as this summation from one particularly candid contributor, reflecting on what she, as a psychiatrist, might represent: ‘I am very clearly a middle class professional White woman who is sitting here telling [the patient] what is happening.’

But who was this woman? Indeed, who were any of these interviewees? Their names are listed in the acknowledgements but not alongside any of their own contributions, an editorial device which purports to leave us free to explore the book in our own way. I found this troubling, untethering, as if I were experiencing these voices in an alternative space. It felt more in service to the interviewees, in defining the boundaries of the reader-writer relationship. It reminded me a little of the dynamic in clinic: you might be able to learn something about a psychiatrist, but not on your own terms. A lot will also be held back.

